# The Evolving Role of the Endoscopic Endonasal Transplanum–Transtuberculum Approach in the Management of Craniopharyngiomas: A Systematic Review of Outcomes, Reconstruction, and Surgical Evolution

**DOI:** 10.3390/jcm15083072

**Published:** 2026-04-17

**Authors:** Omar Alomari, Ali Ozan Yazıcı, Tuğçe Gültepe Zorlu, Aylin Ateş, Cem Çelik, Beyzanur Güney, Gulpembe Bozkurt

**Affiliations:** 1 Hamidiye International School of Medicine, University of Health Sciences, 34668 Istanbul, Türkiye; beyzaguneyst@gmail.com; 2 Department of Otorhinolaryngology, University of Health Sciences, Prof. Dr. Cemil Taşçıoğlu City Hospital, 34384 Istanbul, Türkiye; aliozanyazici@gmail.com (A.O.Y.); tgultepe9797@gmail.com (T.G.Z.); aylinates11221@gmail.com (A.A.); gptalayhan@gmail.com (G.B.); 3 Gaziantep City Hospital, 27470 Gaziantep, Türkiye; ceemcelik1@gmail.com

**Keywords:** craniopharyngioma, EEA, transtuberculum, transplanum, skull base reconstruction

## Abstract

**Background:** Craniopharyngiomas are histologically benign but clinically aggressive epithelial tumors that pose significant surgical challenges due to their proximity to the hypothalamic–pituitary axis. While traditional transcranial approaches are well-established, the endoscopic endonasal transplanum–transtuberculum approach (EETTA) has emerged as a specialized corridor offering direct midline access. This systematic review evaluates the surgical efficacy, functional outcomes, and complication profiles of the EETTA over two decades of clinical evolution. **Methods:** Following PRISMA guidelines, a systematic search was conducted across five databases. Eligible studies included patients of all ages undergoing EETTA for craniopharyngioma. Data on the extent of resection (EOR), visual and endocrine outcomes, and CSF leak rates were extracted. Quality was assessed using NIH/JBI tools, and evidence was graded via AANS/CNS criteria. **Results:** Fifty-one studies (677 patients) were included. The cohort had a mean age of 43.4 years and predominantly suprasellar tumors (mean diameter 2.9–4.0 cm). Gross total resection (GTR) was achieved in 66.4% of cases (364/548). Postoperative visual improvement was reported in 79.8% of patients, while 7.1% experienced deterioration. Endocrine morbidity remained significant, with 120 patients developing new-onset diabetes insipidus and 105 developing new anterior pituitary deficits. The pooled CSF leak rate was 9.2%, with contemporary series frequently reporting 0% due to advanced multilayer reconstruction with nasoseptal flaps. The pooled recurrence rate was 7.8% over a mean follow-up of 37.4 months. **Conclusions:** The EETTA is a safe and effective primary strategy for suprasellar and retrochiasmatic craniopharyngiomas, offering more favorable visual outcomes and resection rates in this specific cohort. While endocrine dysfunction remains a pervasive challenge inherent to the tumor’s location, technical refinements in skull base reconstruction have successfully addressed historical concerns regarding CSF leaks. These findings support the use of the EETTA at high-volume centers with multidisciplinary expertise.

## 1. Introduction

Craniopharyngiomas are rare epithelial tumors of embryologic origin arising from remnants of Rathke’s pouch [1]. These tumors typically develop along the pituitary–hypothalamic axis. They frequently involve critical structures such as the optic chiasm, pituitary stalk, and hypothalamus. Additionally, they often abut the basilar and internal carotid arteries [2]. Although histologically benign, craniopharyngiomas are associated with substantial postoperative recurrence rates (even following gross total resection) largely due to their intimate anatomical relationship with the hypothalamic–pituitary axis and their locally infiltrative growth pattern, which can mimic aggressive biological behavior [3].

Traditionally, a variety of transcranial approaches—including pterional, orbitozygomatic, subfrontal, frontobasal interhemispheric, and transpetrosal routes—have been employed for surgical resection of craniopharyngiomas [4,5,6,7]. While these approaches provide wide exposure, they are often associated with brain retraction and limited inferior midline visualization, particularly for suprasellar lesions, thereby contributing to surgical morbidity [8]. These limitations have driven the development and increasing adoption of alternative minimally invasive strategies.

In this context, endoscopic endonasal approaches (EEAs) have emerged as an important surgical paradigm, driven by advances in endoscopic visualization, anatomical understanding, and surgical instrumentation. However, successful application of EEAs depends not only on effective tumor resection but also on reliable skull base reconstruction. From an otolaryngologic perspective, the endonasal corridor constitutes an active surgical field in which meticulous mucosal management, preservation of vascularized flaps, and reconstruction strategy are integral to operative success.

**Figure 1:**
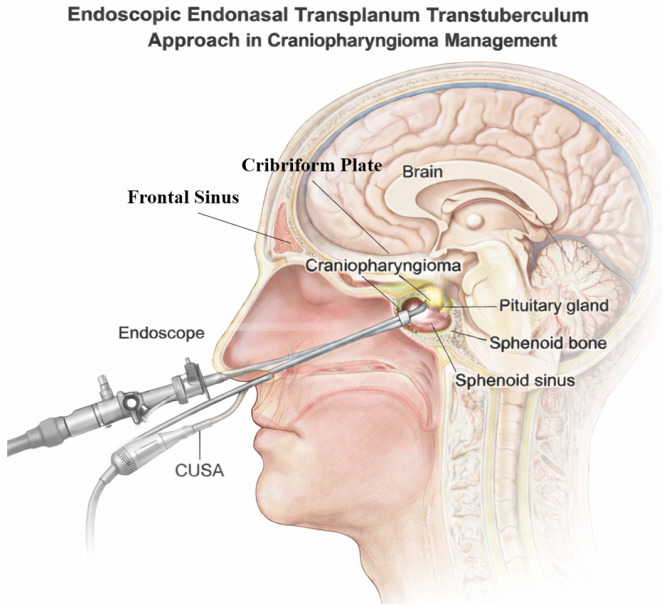
Schematic representation of the extended endoscopic endonasal–transplanum transtuberculum approach. Surgical corridor and anatomical landmarks for the management of suprasellar craniopharyngiomas. This sagittal illustration demonstrates the extended endoscopic endonasal approach (EEA), specifically the transplanum–transtuberculum corridor. The endoscope and Cavitron Ultrasonic Surgical Aspirator (CUSA) are shown traversing the sphenoid sinus to access a craniopharyngioma located in the suprasellar region.

The endoscopic endonasal transplanum–transtuberculum approach (EETTA) provides direct midline access to suprasellar and retrochiasmatic craniopharyngiomas, facilitating early optic apparatus decompression and inferior-to-superior tumor dissection (Figure 1). However, the extended bony and dural defects created at the level of the planum sphenoidale and tuberculum sellae are associated with an increased risk of high-flow cerebrospinal fluid (CSF) leakage, which historically limited the broader adoption of expanded endonasal approaches for suprasellar lesions [9].

The introduction of the vascularized nasoseptal flap (NSF) has fundamentally transformed skull base reconstruction in endoscopic surgery [10]. By providing robust, well-vascularized coverage of large anterior skull base defects, the NSF has been shown to significantly reduce postoperative CSF leak rates and associated infectious complications. In the context of the EETTA, meticulous flap design, preservation of the posterior septal artery pedicle, and multilayer reconstruction techniques are critical determinants of surgical success, underscoring the central role of otolaryngology expertise in minimizing approach-related morbidity.

Despite the availability of multiple surgical approaches for craniopharyngioma resection, selection of the optimal strategy remains challenging. Several authors have advocated anatomically tailored approaches based on tumor-specific relationships with the diaphragma sellae, third ventricle, optic chiasm, and pituitary stalk [7,11,12]. While transcranial approaches are widely accepted for intraventricular craniopharyngiomas and lesions with significant subfrontal or middle cranial fossa extension, and endoscopic endonasal approaches are generally favored for predominantly intrasellar tumors [13,14,15,16,17], the optimal management of intra-suprasellar and suprasellar craniopharyngiomas remains controversial.

In light of the ongoing debate regarding the optimal surgical management of intra-suprasellar and suprasellar craniopharyngiomas, a comprehensive synthesis of existing evidence is warranted. The aim of the present study is to perform a systematic review of the published literature to evaluate the surgical role and clinical outcomes of the EETTA and its extended variants. By utilizing this structured framework, the review seeks to assess the extent of resection (GTR, NTR, and STR), visual and endocrine outcomes, and approach-related complications, with a primary focus on CSF leak rates, to provide a more nuanced understanding of the EETTA role within contemporary, multidisciplinary craniopharyngioma surgery.

## 2. Methods

The systematic literature review was designed to evaluate the surgical role and clinical outcomes of the endoscopic endonasal transsphenoidal transplanum–transtuberculum approach in the management of craniopharyngiomas. This study followed the Preferred Reporting Items for Systematic Reviews and Meta-Analyses (PRISMA 2020) guidelines to maintain methodological transparency [18]. The PRISMA 2020 checklist is provided in the Supplementary Materials (Table S1). The review protocol was prospectively registered in the International Prospective Register of Systematic Reviews (PROSPERO) under ID: CRD420261293159.

### 2.1. Search Strategy

A comprehensive literature search was conducted across five electronic databases: Web of Science, MEDLINE (via PubMed), Scopus, Embase, and the Cochrane Library. All eligible studies published from database inception through 10 February 2026, were considered for inclusion. The search strategy utilized terms such as “Endoscopic Endonasal Transplanum Transtuberculum Approach,” “Extended Endoscopic Transsphenoidal Approach,” and “Endoscopic Endonasal Transtubercular-Transclival Approach.” These terms were combined using Boolean operators (“OR” and “AND”) in accordance with the Cochrane Handbook for Systematic Reviews [19]. Manual screening of reference lists from relevant reviews and included studies was also performed. Detailed database-specific search strategies are provided in Table S2.

### 2.2. Eligibility Criteria

This review included studies involving patients with a radiologically and/or pathologically confirmed diagnosis of craniopharyngioma. Eligible studies encompassed a broad range of study designs, including randomized controlled trials (RCTs), prospective and retrospective cohort studies, case series, and case reports. The target population consisted of human subjects of any age or sex who underwent surgical management specifically via the EETTA or its variants. Studies were required to report data on at least one of the following primary or secondary outcomes: extent of resection (GTR, NTR, STR, or partial), visual outcomes, endocrine status including new-onset diabetes insipidus or panhypopituitarism, and postoperative complications such as CSF leak rates and follow-up duration. Studies were excluded if they were reviews, editorials, technical notes without patient-level data, or conference abstracts lacking original clinical data. Animal studies, in vitro experiments, and non-human research were also excluded. Articles published in languages other than English were excluded unless a complete and reliable translation was available.

### 2.3. Study Selection and Data Extraction

After removal of duplicate records using EndNote software (version 22), two independent reviewers screened the titles and abstracts for relevance. Two additional reviewers according to the predefined inclusion and exclusion criteria then independently assessed full-text articles of potentially eligible studies. Any disagreements at either stage were resolved through discussion and consensus among the review team.

Two reviewers using a pre-piloted, standardized Microsoft Excel data extraction form specifically developed for this review performed data extraction independently. A third reviewer subsequently crosschecked the extracted data to ensure accuracy and consistency. Outcome data were collected as events or continuous variables, with totals, means, and standard deviations reported where available.

### 2.4. Risk of Bias Assessment

Two independent reviewers (O.A., B.G.) assessed the risk of bias in the included studies. The case reports and case series were evaluated with the Joanna Briggs Institute (JBI) critical appraisal tool, which comprises eight questions assessing the methodological quality of a study and the extent to which it addresses potential bias in its design, conduct, and reporting [20]. In addition, we used the NIH tool to assess the quality of cohort and cross-sectional studies, which consisted of 14 questions related to the research methodology [21]. Each question is answered with yes, no, or unclear. Any disagreements in quality assessment were mediated by a third author.

### 2.5. Grading of Evidence and Levels of Recommendations

The included studies were assessed for class of evidence and strength of recommendations according to the grading criteria of the American Association of Neurological Surgeons and the Congress of Neurological Surgeons (AANS/CNS) [22]. Two independent reviewers (O.A., B.G.) evaluated each study, and disagreements were resolved via discussion. The class of evidence (Class I, II, or III) was determined based on study design, sample size, outcome measures, and follow-up data. The corresponding strength of recommendations (Level I–III) was then assigned according to the highest level of supporting evidence.

### 2.6. Statistical Analysis

Descriptive statistics summarized the pooled data, where continuous variables like age and tumor diameter were reported as means or medians with ranges. Categorical variables, including presenting symptoms, resection rates, and visual improvement, were reported as frequencies and percentages. All statistical analyses and figure generation were conducted using R software (Version 4.3.1).

A formal quantitative meta-analysis was not performed due to the significant clinical and methodological heterogeneity of the included literature, which primarily consists of retrospective series and case reports. Consequently, consistent with PRISMA 2020 guidelines for descriptive syntheses, formal heterogeneity testing and publication-bias assessments were omitted to avoid statistical over-interpretation of non-comparative data.

## 3. Results

### 3.1. Baseline Characteristics of Included Studies and Patients

**Figure 2:**
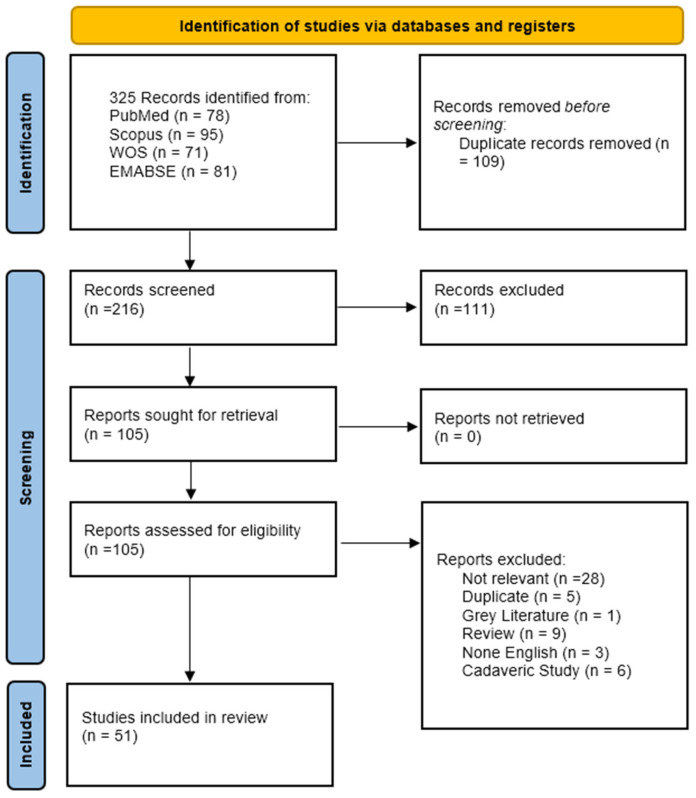
PRISMA flow diagram showing the detailed selecting and selection process of the included articles.

This systematic review included a total of 51 studies, representing a cumulative cohort of 677 patients (Figure 2) [12,23,24,25,26,27,28,29,30,31,32,33,34,35,36,37,38,39,40,41,42,43,44,45,46,47,48,49,50,51,52,53,54,55,56,57,58,59,60,61,62,63,64,65,66,67,68,69,70,71,72]. The distribution of study designs and patient characteristics across the literature is summarized in Table 1. The analyzed literature spans exactly two decades, from 2006 to 2026, reflecting a steady evolution in surgical reporting and technique. The evidence base is predominantly composed of case reports (n = 30) and retrospective cohort studies (n = 9), alongside case series (n = 10) and prospective cohort studies (n = 2).

Geographically, the data reflects a global interest in these clinical outcomes, with the highest volume of research originating from the United States (n = 14), Italy (n = 7), and Japan (n = 6). Significant large-scale data was also contributed by centers in Russia (n = 2), Turkey (n = 2), and Ireland (n = 3). The patient population spanned a broad age demographic, ranging from 23 months to 80 years, with a calculated weighted mean age of 43.37 years. Across the 51 included studies, the patient cohort displayed a nearly even sex distribution with 321 females (47.4%) and 303 males (44.8%), while sex was not specified for 53 patients (7.8%).

### 3.2. Quality, Risk of Bias, and Level of Evidence Assessment

The methodological quality of the included studies was evaluated using the NIH/JBI Quality Assessment tools. The majority of the literature demonstrated high methodological rigor during the reporting of the data, with 40 studies categorized as High Quality: [12,23,24,25,26,27,28,33,34,35,36,38,39,40,41,42,43,44,45,47,48,49,50,51,52,53,54,55,56,57,58,59,60,61,62,63,64,65,66,67,68,69,70,71]. Studies categorized as Fair Quality (n = 7) generally provided valuable clinical insights but lacked specific longitudinal data or granular demographic breakdowns; these include: [30,31,37,40,46,72]. A small minority of studies (n = 2) were classified as Low Quality due to limitations in data completeness or sample size [29,32]. A detailed breakdown of the methodological quality assessment and evidence grading for all included studies is provided in Supplementary Tables S3–S5.

The body of evidence was graded according to the AANS/CNS Classification of Evidence, reflecting the predominance of retrospective surgical series in this field. Class II evidence, representing a higher level of moderate certainty through prospective data collection or comparative analysis, was identified in 11 studies: [37,38,40,41,46,48,53,56,68,71,72]. The remaining 39 studies were classified as Class III, constituting the foundational retrospective evidence for endoscopic endonasal skull base surgery: [12,23,24,25,26,27,28,29,30,31,32,33,34,35,36,39,42,43,44,45,47,49,50,51,52,54,55,57,58,59,60,61,62,63,64,65,66,67,68,69,70].

### 3.3. Clinical Presentation, Tumor Location, and Dimensions

Clinical presentation was predominantly characterized by neuro-ophthalmological deficits and endocrine disturbances (Table 2). Visual acuity loss (VAL) was the most frequent presenting symptom, reported in 128 patients, followed by visual field defects (VFD) in 45 cases. Endocrine and hypothalamic dysfunction were also significant, with hypopituitarism affecting 78 patients and hypothalamic-mediated obesity or weight gain observed in 30 individuals. Neurologically, headache was the primary complaint for 58 patients, while more severe manifestations such as cognitive impairment (n = 7) and hydrocephalus (n = 6) were less common but clinically noteworthy.

The anatomical distribution of the tumors was predominantly suprasellar, with the largest cohort classified as Suprasellar (n = 248). A significant number of cases demonstrated involvement of both the sellar and suprasellar compartments (n = 67). Extension into specialized neuroanatomical regions was frequently noted, including retrochiasmatic/retroinfundibular locations (n = 33), preinfundibular/prechiasmatic areas (n = 21), and extension into the third ventricle (n = 20). Less common locations included transinfundibular tumors (n = 13), sphenoid sinus involvement (n = 11), and purely intraventricular lesions (n = 4). A single rare case was identified within Meckel’s cave.

Analysis of 677 patients undergoing endoscopic endonasal resection revealed a heterogeneous range of tumor sizes. The average maximal diameter across the surgical series was approximately 4.0 cm, while the mean of individual patient measurements was slightly lower at 2.9 cm. The typical range for study means spanned from 2.8 cm to 4.6 cm, with absolute dimensions ranging from a minimum of 1.0 cm to a maximum of 6.7 cm. Larger tumor dimensions were closely associated with increased surgical complexity; specifically, series reporting higher averages (4.2–4.6 cm) typically involved tumors with extensive retrochiasmatic and suprasellar extension. Notably, over 60% of tumors in one series exceeded 3 cm in diameter, underscoring the advanced stage of the disease at the time of surgical intervention.

### 3.4. Extent of Resection

Among the cohort of patients with craniopharyngioma where the extent of resection was explicitly documented (n = 548), Gross Total Resection (GTR) was the most frequently achieved surgical outcome. Specifically, GTR was performed in 364 patients, representing a significant majority of the reported surgical interventions. The remaining patients underwent less-than-total resections, which included Near Total Resection (NTR) in 44 cases, Subtotal Resection (STR) in 106 cases, and Partial Resection (PR) in 31 cases. Minimal intervention, categorized as debulking, was restricted to only 3 patients.

### 3.5. Visual and Endocrine Outcomes

Of the 519 patients with documented postoperative visual assessments, the majority experienced significant clinical benefit. Visual improvement was reported in 414 patients (79.8%), while 68 patients (13.1%) remained stable or unchanged compared to their preoperative baseline. Conversely, a minority of 37 patients (7.1%) experienced a worsening or deterioration of their visual status following the procedure.

The endocrine status of the total pooled cohort (n = 676) following endoscopic endonasal resection revealed a complex landscape of hormonal preservation and new-onset morbidity. Of the patients with quantifiable data, 182 individuals were explicitly identified as having new or worsening endocrine dysfunction. When examining specific deficits, 120 patients developed new-onset Diabetes Insipidus (DI), and 105 patients were diagnosed with new hypopituitarism or anterior pituitary deficits. A comprehensive breakdown of individual hormonal axes and a comparison between preoperative status and postoperative outcomes are provided in Table 3.

### 3.6. Cerebrospinal Fluid (CSF) Leak Outcomes

Across the combined cohort of 677 patients who underwent extended endoscopic endonasal approaches for skull-base lesions, the overall pooled CSF leak rate was calculated to be approximately 9.2%. This estimate is derived from 31 studies that provided quantifiable CSF leak data, representing the majority of the total patient population. The reported leak rates demonstrated considerable heterogeneity, ranging from 0% to as high as 38.46%, reflecting variations in surgical experience, tumor characteristics, cranial base defect sizes, and reconstruction techniques employed across different centers.

The highest leak rate was reported in an early series of midline skull-base lesions, with 38.46% (5 of 13 patients) [27]. The largest cohort in this analysis reported a rate of 8.8% (12 of 136 patients) using a sandwich gasket-seal multilayer reconstruction [37]. Several contemporary series reported zero CSF leaks [33,34,49,50,51,54,55,57,58,59,61,63,65,66,69], likely reflecting the adoption of advanced multilayer closure techniques incorporating vascularized pedicled nasoseptal flaps, fascial grafts, and lumbar drainage protocols. The majority of clinically significant leaks required surgical revision, with many series reporting successful repair using endoscopic multilayer reconstruction techniques, often incorporating autologous tissues such as adipose tissue, fascia lata, bone, and vascularized mucosal flaps, frequently supplemented by lumbar drainage.

Across the series that reported on surgical revisions, a total of 29 patients required repeat operations for postoperative CSF leaks, highlighting the need for additional intervention despite initial skull base repair. The largest cohorts came from Fomichev et al., where all 12 patients with leaks underwent reoperations [37], and Guk and Chukov, where all 8 patients were reoperated using multilayer plasty with autologous tissues, artificial sealing materials, and lumbar drainage for intracranial pressure control [56]. Frank et al. described two revisions where dislocated mucoperiosteum was addressed with a new reconstruction using adipose tissue, bone, and flap repositioning, followed by lumbar drainage [23]. Other successful endoscopic revisions were reported by Divitiis et al. (3 patients total) [12,24] and Cavallo et al. (1 patient) [26]. Additional repairs, though with unspecified techniques, were noted by Sweeney et al. (2 patients) [46] and Tosaka et al. (1 patient) [52]. Collectively, these cases demonstrate that revision surgery—typically employing multilayer techniques, autologous tissues, and lumbar drainage—is consistently effective in definitively managing postoperative CSF leaks.

### 3.7. Follow-Up and Recurrence Analysis

Based on the available data from the cohort of 677 patients, the pooled mean follow-up duration was approximately 37.4 months (3.1 years), derived from 15 studies with quantifiable data. Follow-up periods were highly variable: the shortest mean was 7.2 months [40], while the longest extended to 79.1 months [68], with individual study ranges spanning from 2 to 104 months [38,53].

During this period, the pooled recurrence rate was calculated to be approximately 7.8%, based on 22 studies providing quantifiable data. Recurrence rates were similarly variable, reflecting differences in follow-up duration, tumor characteristics, and extent of resection. The highest rate was 20% (3/15) [38], while the series by Tosaka et al. documented 2 reoperations and 4 small recurrences treated with radiotherapy [52]. Several studies reported no recurrences [23,33,34,37,49,51,54,61,63,65,66,69,70,71]. Other notable rates included 6.5% [68], and isolated cases of recurrence or progression requiring intervention were noted by Alalade et al. [41] and Sweeney et al. [46].

## 4. Discussion

This systematic review synthesizes two decades of evolving clinical experience with the EETTA for craniopharyngioma, encompassing one of the largest pooled patient cohorts reported in the contemporary literature. The pooled data suggest that the EETTA can achieve gross total resection in the majority of appropriately selected patients, with the preponderance of operated individuals experiencing meaningful and durable visual improvement. The pooled recurrence rate observed across the study period is encouraging; however, given that craniopharyngiomas are prone to late recurrence, these findings primarily reflect early-to-mid-term oncological adequacy rather than definitive long-term control. The CSF leak rate, while historically the principal criticism of extended endonasal surgery, has declined substantially at high-volume centers that have adopted advanced multilayer reconstruction techniques including vascularized pedicled nasoseptal flaps.

Endocrine morbidity, particularly new-onset diabetes insipidus and anterior pituitary dysfunction, remains a significant consequence of surgery regardless of approach, reflecting the intimate anatomical relationship between these tumors and the hypothalamic-pituitary axis rather than a deficiency intrinsic to the endonasal route. Critically, postoperative hormonal status is multifactorial and depends heavily on tumor-specific characteristics, including the degree of pituitary stalk involvement and the density of tumor adherence to the third ventricular floor. Furthermore, the risk of new-onset deficits is often a direct trade-off for the extent of resection, as aggressive attempts at gross total resection in cases of firm adherence can exacerbate damage to the fragile infundibular structures. Consequently, a multidisciplinary approach integrating endocrinological management is indispensable. Taken together, the results support that the EETTA, in experienced hands, constitutes a safe, effective, and anatomically rational strategy for suprasellar and retrochiasmatic craniopharyngiomas while continuing to highlight areas where further refinement is required.

Craniopharyngiomas are rare, histologically benign epithelial tumors of World Health Organization (WHO) Grade I classification, arising from remnants of Rathke’s pouch within the sellar and parasellar region [1]. Despite their benign classification, craniopharyngiomas occupy a uniquely challenging niche in neurosurgical oncology. Their intimate anatomical proximity to the optic pathways, hypothalamus, third ventricle, pituitary gland, and the internal carotid arteries renders them capable of producing profound neurological, visual, endocrine, and hypothalamic dysfunction even before reaching large dimensions [73].

The clinical burden of craniopharyngioma far exceeds that of most benign brain tumors. Patients face a nearly threefold increase in mortality driven by a fivefold rise in cerebrovascular death, along with high rates of diabetes, stroke, and severe infection [74]. Hypothalamic damage often leads to debilitating obesity with metabolic and psychosocial consequences [75]. Most patients present with visual deficits and hypopituitarism, requiring lifelong hormone replacement [75]. Neuropsychological impairment, especially in children, further diminishes quality of life [75]. These findings underscore the need for aggressive, comprehensive management and justify surgical intervention even in stable patients, despite the tumor’s benign classification.

Open transcranial microsurgery has historically constituted the foundation of craniopharyngioma management. The introduction of the operative microscope, pioneered in part by Yasargil and colleagues, dramatically expanded the resection envelope and remains one of the landmark advances in skull base surgery [76]. A diverse repertoire of transcranial corridors has been refined over several decades to address heterogeneous tumor configurations. These include pterional, bifrontal, and orbitozygomatic approaches. Additionally, transcallosal or transpetrosal routes are employed for intraventricular or deeply retrochiasmatic lesions [77]. Each approach offers genuine oncological advantages in specific anatomical configurations, particularly for tumors with significant lateral extension, retrosellar or interpeduncular involvement, or predominantly intraventricular growth that exceeds the reach of the endonasal corridor [77].

However, the advantages of transcranial surgery are counterbalanced by significant procedure-related morbidity, even with refined microsurgical technique. All transcranial approaches require some degree of brain retraction or manipulation of neurovascular structures [78]. This can lead to frontal lobe contusions, venous thrombosis, and edema. Manipulation of the optic apparatus carries a tangible risk of postoperative visual deterioration [79], and access to the retrochiasmatic space and hypothalamus is generally limited [79]. In large series, gross total resection rates range from 50 to 90%, but this is accompanied by high endocrine morbidity, including diabetes insipidus (40–80%) and anterior pituitary insufficiency [80]. Hypothalamic damage, the primary driver of poor long-term quality of life, is directly related to surgical manipulation in these critical zones [81]. Tumor recurrence rates range from 12% to over 25% at five years [79,80,81]. These data underscore that surgery-related morbidity remains substantial, justifying the search for less traumatic alternatives.

The development of endoscopic endonasal surgery marked a paradigm shift in managing sellar and parasellar pathology. Building on the transsphenoidal approach pioneered in the early twentieth century and later advanced with the introduction of the operating microscope, the fully endoscopic technique was popularized in the late 1990s and early 2000s. Compared to the microscopic approach, the endoscope offered a panoramic wide-angle view of the operative field, enhanced illumination, angled visualization, and enabled a two-surgeon four-hand technique without the constraints of a nasal speculum. For sellar lesions such as pituitary adenomas, these advantages translated into higher rates of gross total resection and lower complication rates at experienced centers.

The extension of the endoscopic endonasal approach to suprasellar, retrochiasmatic, and parasellar pathology marked the next evolutionary step. The expanded endoscopic endonasal approach, encompassing a spectrum of anatomically defined corridors including the transplanum–transtuberculum route, the transcribriform approach, and the far-medial transclival approaches, extended the reach of endonasal surgery to lesions previously accessible only through craniotomy [25]. For craniopharyngiomas specifically, the extended transsphenoidal approach to suprasellar tumors was described by de Divitiis, Cappabianca, and Cavallo from the Naples group in the mid-2000s, and independently developed by the Pittsburgh group under Gardner and Fernandez-Miranda, who systematically elaborated the endoscopic anatomy of the pituitary corridor and its extensions [12,32]. These early series established both the feasibility and the limitations of the technique, drawing attention particularly to the high rate of postoperative CSF leaks characterizing the early learning curve before advanced skull base reconstruction strategies were universally adopted.

Among the extended endonasal corridors, the transplanum–transtuberculum approach occupies a position of particular relevance for craniopharyngioma surgery, as it provides the most direct midline access to the prechiasmatic, retrochiasmatic, and suprasellar spaces [32]. The approach entails sequential removal of bone beginning from the posterior wall of the sphenoid sinus and extending anteriorly over the planum sphenoidale and across the tuberculum sellae, opening a bone window that allows direct visualization of the prechiasmatic sulcus, the undersurface of the optic chiasm, the chiasmatic cistern, the pituitary stalk, and the retrochiasmatic compartment [32,36]. This trajectory approaches the tumor from below and behind the optic apparatus. By exploiting the corridor between the optic nerves, it provides direct exposure of the chiasmatic undersurface and hypothalamus. Crucially, this is achieved without displacing these structures from the “outside-in [10]. This anatomical advantage is fundamentally different from the approach geometry of most transcranial corridors, which must negotiate the optic apparatus from above and in front, often requiring instrument passage beneath the chiasm to access retrochiasmatic tumor [10].

The operative technique comprises two principal phases. The nasal phase involves bilateral endonasal access using a four-hand binostril technique, harvest of a vascularized pedicled nasoseptal flap for reconstruction, bilateral sphenoidotomy, and full exposure of the posterior sellar wall, tuberculum sellae, and planum sphenoidale [10]. The cranial phase proceeds with sequential bony removal to create an adequate bone window, followed by dural opening, tumor decompression, and excision using microdissection principles adapted for the bimanual endoscopic technique. Angled telescopes enable direct visualization of retrochiasmatic and hypothalamic surfaces, as well as tumour extensions into the third ventricle and interpeduncular cistern [34]. Skull base reconstruction employs a multilayer approach incorporating an inlay fascial graft, rigid buttressing material, and the vascularized nasoseptal flap as a waterproof outer layer—a strategy that has dramatically reduced postoperative CSF leak rates compared to earlier techniques [10,82].

Several classification systems have been developed to guide the selection of this approach versus alternative transcranial routes. The QST classification, for example, distinguishes infrasellar/subdiaphragmatic (Q-type), subarachnoidal (S-type), and pars tuberalis (T-type) craniopharyngiomas based on anatomical origin and growth pattern, providing a useful framework for selecting the optimal surgical corridor [83]. Broadly, the transplanum–transtuberculum endonasal approach is considered most advantageous for strictly midline tumors with pre- or retrochiasmatic supradiaphragmatic extension that lack significant lateral involvement of the cavernous sinuses or middle cranial fossa, and for which the relationship to the pituitary stalk can be clearly defined on preoperative imaging [32].

Multiple comparative studies and meta-analyses have systematically examined the relative merits of the endoscopic endonasal approach and transcranial surgery for craniopharyngioma. The visual outcomes documented in this review, with the large majority of patients achieving postoperative improvement, are consistent with and favorable relative to those reported in published comparative series [78,84]. A landmark systematic review and meta-analysis by Na et al. demonstrated that EEA was associated with a significantly higher likelihood of postoperative visual improvement compared with transcranial approaches, with an incidence of visual improvement approaching 60.7% for EEA versus 32.7% for transcranial surgery, alongside a significantly lower likelihood of postoperative visual deterioration [85]. Similarly, Figueredo et al., in a ten-year systematic review and meta-analysis, reported favorable rates of visual improvement and gross total resection in the EEA group compared to historical transcranial cohorts, though these findings remain subject to the selection biases inherent in non-randomized studies [85]. The recurrence rate of approximately 7.8% documented in this review compares favorably with pooled transcranial series reporting recurrence rates of twenty percent or higher at comparable follow-up intervals, concordant with the finding of Figueredo et al. who showed a fifteen percent recurrence rate for EEA versus twenty-one percent for transcranial surgery [85].

The study by Fan et al. using the QST classification in a large comparative series of 315 patients confirmed that for appropriately selected subarachnoidal and subdiaphragmatic craniopharyngiomas, EEA was associated with significantly higher gross total resection rates, lower recurrence rates, and lower new hypopituitarism rates compared with the transcranial approach [83]. A systematic review specifically examining hypothalamic morbidity found that while endocrinological outcomes were broadly comparable between approaches, EEA was associated with less postoperative visual deterioration and lower tumor recurrence, with transcranial approaches carrying a significantly higher recurrence risk [86]. The single-institution comparative series by Nie et al. similarly confirmed that EEA achieves more favorable gross total resection rates, better postoperative visual recovery, lower rates of hypopituitarism and diabetes insipidus, and lower recurrence rates relative to transcranial surgery [78].

The CSF leak rate of approximately 9.2% observed in this pooled analysis, while higher than that typically reported for transcranial approaches, must be interpreted within its proper historical and technical context. Early endonasal extended series from the mid-2000s reported CSF leak rates of thirty to forty percent, reflecting the absence of vascularized reconstruction strategies [37]. The introduction of the pedicled nasoseptal flap by Hadad and colleagues in 2006 and its subsequent adoption at dedicated skull base centers have progressively reduced leak rates; contemporary high-volume series report rates of five percent or less, and several centers in this review reported zero postoperative CSF leaks [37,82]. The reliability of these modern endonasal endoscopic repair strategies is further supported by broader skull base literature, such as Ismaiel et al. (2021), who demonstrated a 100% success rate in the endoscopic repair of complex dural defects with a minimal morbidity profile [87]. Consequently, the residual heterogeneity in pooled leak rates reflects variable institutional experience and the temporal shift toward advanced multilayered closure rather than an inherent deficiency of the surgical corridor itself.

It must be acknowledged that the high rates of GTR and low CSF leak rates reported in this review likely reflect surgeon and center expertise bias. Most included studies originate from high-volume skull base centers with significant endoscopic experience. Consequently, these outcomes may not be immediately generalizable to lower-volume centers or surgeons in the early stages of the EETTA learning curve.

Surgery remains the cornerstone of treatment for most craniopharyngiomas, with radiotherapy reserved for incomplete resections, recurrence, or when surgical risk is excessive [88]. Stereotactic radiosurgery (SRS) offers tumor control in carefully selected patients with small solid remnants distant from the optic apparatus; a recent meta-analysis reported pooled control rates of 76% [89]. Fractionated stereotactic radiotherapy (FSRT) provides comparable efficacy with favorable toxicity profiles [89]. Although conventional fractionated radiotherapy may offer better long-term progression-free survival, complication rates are broadly similar across modalities [89].

The recurrence rate of 7.8% documented in this review compares favorably with the historically high recurrence rates reported after incomplete transcranial surgery without adjuvant radiotherapy, and approaches the oncological control achieved with combined surgery and radiotherapy strategies, suggesting that the high gross total resection rates achievable via the endonasal corridor may reduce or eliminate the need for postoperative radiation in a meaningful proportion of patients [85]. However, the variable and relatively short follow-up duration across the pooled cohort limits the ability to draw definitive conclusions regarding late recurrence patterns, which remain a hallmark of this disease. Nevertheless, the residual endocrine morbidity observed in this pooled series underscores that surgery alone, regardless of approach, cannot fully preserve hypothalamic-pituitary axis function in all patients, and that a multidisciplinary approach integrating endocrinological management is indispensable.

The clinical implications of this systematic review are several and significant. First, it provides one of the largest pooled evidence bases specifically examining the EETTA for craniopharyngioma, offering a level of statistical power that individual institutional series cannot achieve given the rarity of the disease. Second, this synthesis suggests that the EETTA achieves gross total resection in the majority of appropriately selected patients, with a visual improvement rate approaching eighty percent, an outcome of paramount importance given that neuro-ophthalmological deficits are the most common and most debilitating presenting manifestation. Third, it illustrates a trend where with the adoption of vascularized flaps, CSF leak rates may become comparable to those of transcranial surgery, supporting a clear technique-dependent rather than approach-dependent relationship with this complication. Fourth, the recurrence rate documented over a mean follow-up exceeding three years compares favorably with historical series, supporting the oncological adequacy of the endonasal corridor as a definitive surgical strategy. Fifth, the endocrine outcomes data reinforce the recognition that hypothalamic-pituitary morbidity is an inherent consequence of craniopharyngioma surgery at this location regardless of approach, and that realistic counseling of patients regarding the likelihood of lifelong endocrine replacement therapy is a necessary component of the preoperative discussion. Finally, the global geographic distribution of reporting centers attests to the widespread adoption of this technique and supports its increasing recognition as the approach of choice for midline suprasellar craniopharyngiomas at experienced skull base centers.

Beyond surgical technique, the modern management of craniopharyngioma has evolved into a highly specialized multidisciplinary framework. A primary shift in this paradigm is the adoption of hypothalamus-sparing strategies, which prioritize the preservation of metabolic and neurocognitive function over the historical mandate for aggressive gross total resection [90]. In cases where dense tumor adherence to the third ventricular floor is encountered, a strategic subtotal resection followed by adjuvant radiotherapy (such as proton beam therapy or fractionated stereotactic radiotherapy) is increasingly utilized to achieve long-term tumor control while minimizing devastating hypothalamic obesity [90,91]. Furthermore, subtype-specific treatment considerations are emerging as a critical component of care; for instance, the identification of BRAF V600E mutations in papillary craniopharyngiomas has opened avenues for targeted neoadjuvant or adjuvant pharmacotherapy [91]. Ultimately, the successful management of these complex lesions requires a lifelong collaborative effort between neurosurgeons, endocrinologists, radiation oncologists, and neuropsychologists to balance oncological control with the preservation of functional quality of life.

This systematic review offers a comprehensive, 20-year synthesis of EETTA outcomes across 51 studies and 14 countries, providing high-quality, generalizable data graded by the AANS/CNS framework. Its multidimensional analysis of resection, vision, and recurrence offers a robust overview of surgical performance. However, the reliance on Class III-II retrospective data introduces inherent selection bias and significant reporting heterogeneity. While 40 studies demonstrated high methodological quality within their design frameworks, the absence of Class I evidence inherently limits the strength of definitive clinical recommendations.

Furthermore, the inclusion of numerous case reports and small retrospective series introduces substantial publication and selection bias, where positive outcomes are likely overrepresented. The EETTA is typically reserved for midline, suprasellar, or retrochiasmatic lesions with favorable anatomy, whereas tumors with significant lateral extension or complex neurovascular involvement are often diverted to transcranial corridors. Consequently, the high rates of gross total resection and visual recovery documented here may reflect this strategic case selection rather than the inherent superiority of the endonasal approach. The ‘center-effect’ wherein outcomes are driven by highly specialized expertise, further limits the external validity of the pooled data, a factor that should be carefully considered when extrapolating these results to broader surgical practice. Other limitations include a relatively short follow-up period for detecting late recurrences and a lack of data on critical quality-of-life metrics, such as hypothalamic obesity and neurocognitive function.

Furthermore, the absence of concurrent control arms precludes definitive comparative conclusions, highlighting the urgent need for prospective, multi-institutional registries with standardized protocols.

Lastly, the descriptive nature of current craniopharyngioma literature highlights a critical need for more granular, standardized data reporting to facilitate future quantitative meta-analyses. Future studies should move beyond aggregate outcomes and prioritize reporting patient-level data categorized by specific clinical and radiological subgroups. Specifically, there is an urgent requirement for multi-institutional prospective registries that utilize standardized classification systems such as the QST or Kassel classifications, to enable meaningful subgroup analyses based on tumor origin, size, and relationship to the third ventricle. Additionally, future research should aim to perform comparative studies stratified by age group and surgical volume, as these variables remain significant sources of clinical heterogeneity. By adopting a common data element approach to reporting GTR rates, visual field scores, and quality-of-life metrics (such as hypothalamic obesity and neurocognitive function), the neurosurgical community can transition from descriptive syntheses to high-powered, evidence-based comparative trials. This evolution is essential to definitively establish the role of the EETTA relative to transcranial and radiation-based strategies in the modern management of craniopharyngioma.

## 5. Conclusions

This systematic review of 677 patients treated over a twenty-year period (2006–2026) establishes the EETTA as a safe, effective, and anatomically more favorable primary strategy for managing suprasellar and retrochiasmatic craniopharyngiomas. The data demonstrate that the EETTA achieves high rates of gross total resection and better visual recovery compared to traditional transcranial corridors, primarily by providing direct, midline access without the need for brain retraction. While endocrine morbidity remains a persistent and often unavoidable challenge due to the tumor’s intimate relationship with the hypothalamic-pituitary axis, the historical risk of cerebrospinal fluid leaks has been successfully mitigated by the adoption of advanced multilayer reconstruction techniques and vascularized nasoseptal flaps. Consequently, in the hands of experienced multidisciplinary teams at high-volume centers, the EETTA serves as an oncologically adequate and functionally restorative surgical approach for appropriately selected lesions, though long-term prospective registries are still required to fully characterize late recurrence patterns and quality-of-life outcomes.

## Figures and Tables

**Table 1:** Summary of Clinical Characteristics and Surgical Outcomes for Endoscopically Managed Craniopharyngiomas.

Study ID	Year	Study	Country	Sample Size	Age	Sex	Presenting Symptoms	Location	Lesion Diameter	Quality Rating (NIH/JBI)	CNS/AANS Class
Frank et al. [23]	2006	Case Series	Italy	10	Mean 42 (11–61)	4 Males, 6 Females	BT Hemianopi, hypopituitarism, DI	Purely supradiaphragmatic (six patients), with a significant suprasellar component (four patients)	mean diameter of 2.9 cm (range, 1–4 cm)	High Quality	Class III
Divitiis et al. [12]	2007	Case Series	Italy	10	Mean 57.2	6 Males, 4 Females	BT Hemianopi, hypopituitarism, DI	2 intrasuprasellar, 1 suprasellar, 6 suprasellar-intraventricular, 1 purely intraventricular	Not specified	High Quality	Class III
Divitiis et al. [24]	2007	Case Series	Italy	7	Mean 46.4	4 Male, 3 Female		Suprasellar lesions.	Not specified	High Quality	Class III
Laufer et al. [25]	2007	Case Series	USA	4	Mean 52.25	Not specified	visual loss/hyperphagia, DI, hypopituatizm	Suprasellar lesions.	mean 3375	High Quality	Class III
Cavallo et al. [26]	2009	Case Series	Italy	22	Mean 49.4 (18–80)	15 Male/7 Female	BT hemianopsia, decreased VA, PHP, DI, obesity, consciousness impairment	12 Suprasellar, 9 Intra-suprasellar, 1 Meckel cave	1.4–4.5 cm	High Quality	Class III
Ceylan et al. [27]	2009	Case Series	Turkey	2	Mean 49	2 Females	Not specified	Prechiasmatic with intrasellar cystic components	not specified	High Quality	Class III
Saeki et al. [28]	2010	Case Report	Japan	1	43	Male	left visual field defect.	Suprasellar lesions.	1.5 cm	High Quality	Class III
Kalinin et al. [29]	2011	Case Series	Russia	27	Not specified	Not specified	Not specified	Suprasellar lesions.	mean 4.2 cm	Low Quality	Class III
Ferroli et al. [30]	2012	Case Report	Italy	1	40	Male	chronic headache in case one and for headache, postural instability, visual impairment in left eye, weight increase, polydipsia, and polyuria in case two.	Midline suprasellar	not specified	Fair Quality	Class III
Kenning et al. [31]	2012	Case Report	USA	1	21	Female	progressive bitemporal hemianopsia.	Suprasellar, retrochiasmatic	Not specified	Fair Quality	Class III
Lui et al. [32]	2012	Case Report	USA	1	21	Female	bitemporal hemianopsia	Suprasellar, retrochiasmatic	Not specified	Low Quality	Class III
De lara et al. [33]	2013	Case Report	USA	1	68	Female	Visual deterioration accompanied by worsening headaches	Suprasellar region	Not specified	High Quality	Class III
Iacoangeli et al. [34]	2014	Case Report	Italy	1	24	Male	Headache, visual deficits and transient diplopia	Sellar region to clivus	Not specified	High Quality	Class III
Ceylan et al. [35]	2015	Case Series	Turkey	16	Mean 30.2 (14–55)	12 Female 4 Male	Not specified	10 preinfundibular, 2 retroinfundibular, Transinfundibular–Retroinfundibular–Preinfundibular 4	Not specified	High Quality	Class III
Sankhla et al. [36]	2015	Case Series	India	15	8–15	9 Female, 6 Males	Headache and diminished vision, isolated cranial nerve deficits, seizures, and altered sensorium.	Retrochiasmatic		High Quality	Class III
Javadpour et al. [38]	2016	Retrospective Cohort	Ireland	11	Median 9 (5–18)	Not specified	Not specified	Not specified	Not specified	Fair Quality	Class II
Fomichev et al. [37]	2016	Retrospective Cohort	Russia	136	Mean 49.3	Female (55.9%), Male (44.1%)	Vision loss (82.4%), Hypothalamic-pituitary dysfunction (54.4%), Headaches (31.6%), Mental impairment (20.6%), Obstructive hydrocephalus (11.8%)	111 suprasellar and 25 intra-extraventricular	4.2 cm	High Quality	Class II
Nishioka et al. [39]	2016	Case Report	Japan	3	Case 1: 65 case 2: 29 case 3: 5	Case 1: Female Case 2: Female Case 3: Male	Case 1: visual disturbance, headache Case 2: Hypogonadism, visual disturbance (bitemporal hemianopsia) Case 3: headache, vomiting, and double vision	Case 1: third ventricle Case 2: third ventricle Case 3: third ventricle	Case 1: 20 mm Case 2: 30 mm Case 3: 38 mm	High Quality	Class III
Wannemuehler et al. [40]	2016	Retrospective Cohort	USA	9	Mean 52.4	Female (33.3)/Male (66.7)	Headache 66.7, Visual disturbance 88.9, Hypopituitarism 33.3	Suprasellar and retrochiasmatic extension	4.6	Fair Quality	Class II
Alalade et al. [41]	2018	Retrospective Cohort	USA	11	Mean: 7.9	Male: 8/Female: 3	visual impairment 6, growth retardation 5, headches 4, somnolence/lethargy 2, cognitive impairment 1, polyuria 1	Sphenoid sinuses were conchal (n = 5), presellar (n = 4) and sellar (n = 2).	1.3 to 41.7 cm^3^	High Quality	Class II
Almedia et al. [42]	2018	Case Report	Canada	1	56	Female	Bitemporal hemianopsia and visual acuity	Suprasellar craniopharyngioma.	Not specified	High Quality	Class III
Liu et al. [43]	2018	Case Report	USA	1	12	Male	Progressive visual loss and panhypopituitarism	Retrochiasmatic in the suprasellar region with third ventricular extension	Not specified	High Quality	Class III
Gomes et al. [44]	2018	Case Report	Brazil	1	72	Male	Bitemporal visual loss, mildly elevated prolactin levels	Suprasellar craniopharyngioma	Not specified	High Quality	Class III
Messerer et al. [45]	2018	Case Report	Switzerland	1	52	Male	Biitemporal hemianopia, bilateral decreased visual acuity	Retrochiasmatic with sellar and parasellar extension	Not specified	High Quality	Class III
Sweeney et al. [46]	2017	Prospective Cohort	Ireland	12	Range 5–18	Not specified	Vision loss: 10	Not specified	Not specified	Fair Quality	Class II
Todeschini et al. [47]	2017	Case Report	United states	1	57	Male	Vision loss	Suprasellar with third ventricle extension (type II Kassam).	Not specified	High Quality	Class III
Gauden et al. [48]	2019	Retrospective Cohort	New Zealand	9	Mean 37.6 (14–68)	Female 3/Male 6	Visual deficit (100%), headache (89%), Panhypopituitarism 67%	Suprasellar 56%, Sellar extension 33%, 3rd ventricle extension 22%	from 1.5 to 3.4 cm	High Quality	Class II
Wang et al. [49]	2019	Case Report	China	1	38	Female	20 years headache; 30 days blurred vision; VF defect and ↓ visual acuity	Suprasellar CP; severely calcified, poorly developed sphenoid sinus; narrow acoma–planum distance and narrow bilateral ICA distance (high-risk anatomy)	1.4 × 1.8 cm	High Quality	Class III
Almeida et al. [50]	2019	Case Report	Canada	1	52	Female	Progressive visual decline and headaches; no hormonal deficiencies	Sellar–suprasellar solid-cystic lesion (CP), mainly preinfundibular, anterior to chiasm; medial to PcomA	Not reported	High Quality	Class III
Liu et al. [51]	2020	Case Report	USA	1	56	Female	slight confusion, progressive headache and visual loss, increased appetite, and weight gain.	Suprasellar retrochiasmatic region	Not specified	High Quality	Class III
Tosaka et al. [52]	2020	Case Series	Japan	19	Mean 48 (9–68)	9 Male, 10 Female	NR (not reported as a structured list)	Suprasellar craniopharyngioma	Mean 28.5 mm (11–45 mm)	High Quality	Class III
Javadpour et al. [53]	2021	Prospective Cohort	Ireland	15	Median 10 (5–18)	9 Male, 6 Female	Visual impairment ± headache; other presentations include vomiting, weight gain, growth retardation/delayed puberty, amenorrhea, DI	Transinfundibular (9/15), preinfundibular (5/15), retroinfundibular (1/15)	Tumor diameters measured SI × W × AP on MRI; examples range 20 × 17 × 15 mm up to 67 × 64 × 51 mm	High Quality	Class II
Ryan et al. [54]	2021	Case Report	USA	1	4	Female	Worsening headaches and vision loss	Not specified	24.3 × 25.3 × 19.5 mm (suprasellar cystic component)	High Quality	Class III
Ohta et al. [55]	2022	Case Report	Japan	1	61	Male	Progressive right-sided vision loss and left-sided visual field loss	Pituitary suprasellar cystic tumor consistent with craniopharyngioma; pituitary stalk amputated for complete tumor removal	26 × 18 × 24 mm	High Quality	Class III
Guk and Chukov [56]	2023	Retrospective Cohort	Ukraine	69	Mean 46.7 (19–73)	60.9% Female, 39.1% Male	Visual disturbances (78.3%), Hypopituitarism (58%), Diabetes insipidus (14.5%)	Supradiaphragmatic (n = 65), Infradiaphragmatic (n = 4)	Not specified as a single mean; majority were cystic-solid (73.9%)	High Quality	Class II
Kamal et al. [57]	2023	Case Report	Uk	1	48	Female	Progressive visual field loss; later orthostatic headache, polyuria, and polydipsia	Suprasellar (cystic recurrence)	Not specified	High Quality	Class III
Khalil et al. [58]	2023	Case Report	France	1	68	Male	Not specified	Supradiaphragmatic and intraventricular extension	Not specified	High Quality	Class III
Shen et al. [59]	2023	Case Report	China	1	41	Female	Not specified	Suprasellar infundibulo-tuberal	Extremely narrow CPC (<5 mm)	High Quality	Class III
Constanzo et al. [60]	2024	Case Report	USA	1	2	Male	Adrenal insufficiency, hypothyroidism, visual field/light perception abnormalities	Tubero-infundibular (Recurrent)	Not specified	High Quality	Class III
Chen et al. [61]	2024	Case Report	China	1	59	Female	Intermittent headache and decreased visual acuity	Suprasellar region (CPG) and Tuberculum sellae (MNG)	Not specified	High Quality	Class III
Vigo et al. [62]	2023	Case Report	USA	1	13	Male	Stunted growth, decreased vision, headaches, low energy	Tuberoinfundibular (suprasellar/retrochiasmatic)	Not specified	High Quality	Class III
Eaton et al. [63]	2024	Case Report	USA	1	23 months	Male	Nystagmus and falls	Sellar and suprasellar	19.3 × 42.1 mm (Volume: 8.7 cm^3^)	High Quality	Class III
Finger et al. [64]	2024	Case Report	USA	1	66	Male	Bitemporal hemianopsia, Erectile dysfunction, Nocturia, Lightheadedness	Suprasellar (Type IV, protruding into 3rd ventricle)	2.9 cm	High Quality	Class III
Matmusayev et al. [65]	2024	Case Report	Japan	1	56	Male	Visual disturbances (L homonymous hemianopia), cognitive dysfunction	Suprasellar and retroinfundibular	48 mm (max)	High Quality	Class III
Moiyadi et al. [66]	2024	Case Report	India	1	Young boy	Male	Polyuria, hypersomnia, lethargy, headache, vision loss	Sellar-suprasellar (extending to 3rd ventricle floor)	Large	High Quality	Class III
Noiphithak et al. [67]	2024	Case Report	Thailand	1	16	Female	Progressive vision loss (decreased VA, bitemporal hemianopsia), Secondary adrenal insufficiency, Hyperprolactinemia	Suprasellar with retrosellar extension	Not specified	High Quality	Class III
Bove et al. [68]	2025	Retrospective Cohort	Italy	61	Mean 51.87	55.7% Male, 44.3% Female	Preoperative visual impairment (85.2%), Pituitary dysfunction (50.8%), Obesity (39.3%), Bitemporal hemianopsia (36%), Visual acuity (32.8%), Panhypopituitarism (21.3%), Hypopituitarism 1 axis (14.8%), Hydrocephalus (8.2%), Panhypopituitarism + DI (8.2%), Headache (6.5%), Consciousness impairment (6.5%), Hemianopsia + Quadrantanopia (4.9%), Bilateral quadrantanopia (4.9%), Hypopituitarism 2 axis (3.3%), DI (3.3%), Unilateral quadrantanopia (3.3%), Memory disturbance (3.2%), Unilateral hemianopsia (1.6%), Amaurosis + Hemianopsia/Quadrantanopia (1.6%)	Two cases (3.3%) were purely intraventricular cps. Most of the lesions (96.7%) presented with various degree of secondary involvement: 22 (36%) involved the SI, 28 (45.9%) the Infundibulum–ventricular chamber, and 9 (14.8%) the SI- Ventricular chamber.	60.7% > 3 cm	High Quality	Class II
Matmusaev et al. [69]	2025	Case Report	Japan	1	48	Male	Visual disturbances and weight gain	Third ventricle	30 mm	High Quality	Class III
Olson et al. [70]	2025	Case Report	USA	1	41	Female	Amenorrhea, weight gain, progressive visual field changes, hypothyroidism, and elevated prolactin levels	Sellar and suprasellar	Large mixed solid and cystic mass	High Quality	Class III
Elshazly et al. [71]	2026	Retrospective Cohort	Egypt	14	Mean 11.1 (5–16)	64.3% Male, 35.7% Female	Visual impairment (100%) and headache (71.4%)	Purely supradiaphragmatic (Anterior skull base)	Mean maximal diameter: 3.6 cm (Range: 2.7–5.1)	High Quality	Class II
Andrade et al. [72]	2023	Retrospective Cohort	USA	166	Median 53.36	47% Male, 53% Female	Not specified	Not specified	Not specified	Fair Quality	Class II

AANS: American Association of Neurological Surgeons; AP: Anteroposterior; BT Hemianopi/Hemianopsia: Bitemporal Hemianopsia; CNS: Congress of Neurological Surgeons; CPC: Craniopharyngeal Canal; CP/CPG: Craniopharyngioma; DI: Diabetes Insipidus; ICA: Internal Carotid Artery; JBI: Joanna Briggs Institute; L: Left; MNG: Meningioma; MRI: Magnetic Resonance Imaging; NIH: National Institutes of Health; NR: Not Reported; PcomA: Posterior Communicating Artery; PHP: Panhypopituitarism; SI: Sella Iurcica (Sellar Involvement) or Superior-Inferior (depending on context of measurement); UK: United Kingdom; USA: United States of America; VA: Visual Acuity; VF: Visual Field; W: Width.

**Table 2:** Presenting Signs and Symptoms Among Patients Undergoing Endoscopic Endonasal Resection of Craniopharyngioma.

Category	Specific Symptom	Frequency (n)
**Neuro-Ophthalmological**	Visual Acuity Loss (VAL)	128
	Visual Field Defects (VFD)	45
	Diplopia	2
	Nystagmus	1
**Endocrine and Hypothalamic**	Hypopituitarism/pituitary dysfunction	78
	Obesity/weight gain	30
	Diabetes insipidus (symptoms)	6
	Growth retardation	6
	Hypogonadism/ED/amenorrhea	3
	Hyperprolactinemia	3
	Adrenal insufficiency	2
	Hypothyroidism	2
	Nocturia	1
**Neurological and General**	Headache	58
	Cognitive impairment	7
	Hydrocephalus	6
	Somnolence/lethargy	4
	Consciousness impairment	4
	Ataxia/falls/postural instability	2
	Nausea/vomiting	1
	Lightheadedness	1
	Low energy	1

**Table 3:** Surgical Outcomes, Complications, and Reconstruction Techniques in Endoscopic Endonasal Resection.

Study ID	Year	GTR/NTR Rate	Visual Outcome	Endocrine Status (New DI/Deficits)	CSF Leak Rate (n/N)	Follow-Up	Recurrence
Frank et al. [23]	2006	7 GTR, 1 STR, and 2 PR	Improved significantly in six out of eight patients	Did not improve	3 cases	Mean 37 months	No
Divitiis et al. [12]	2007	7 GTR, 2 STR, 1 PR	Visual field and/or acuity defect improved except one patient	Did not improve	2 patients	Not specified	Not specified
Divitiis et al. [24]	2007	5 GTR, 2 STR	5 improved 2 unchanged	Not specified	1 patient	Not specified	Not specified
Laufer et al. [25]	2007	Not specified	Improved	DI and panhypopituitarism develop in approximately 70%	1 transient leak	8.5	Not specified
Cavallo et al. [26]	2009	10 GTR, 9 STR, 3 PR	Improved	Did not improve	1 patient (13.6%)	Not specified	Not specified
Ceylan et al. [27]	2009	2 GTR	Not specified	Not specified	5 of 13 (38.46%)	Median: 17.76 months	Not specified
Saeki et al. [28]	2010	Not specified	Not specified	Not specified	Not specified	Not specified	Not specified
Kalinin et al. [29]	2011	70.4% GTR, 29.6% STR	Visual improvement in 13 (48%) patients, one patient (3.7%) permanent worsening of vision	Developed in 7 (26%) cases	3 of 27 (11%)	Not specified	Not specified
Ferroli et al. [30]	2012	GTR	Not specified	Not specified	Not specified	Not specified	Not specified
Kenning et al. [31]	2012	GTR	Not specified	Not specified	Not specified	Not specified	Not specified
Lui et al. [32]	2012	GTR	Not specified	Not specified	Not specified	Not specified	Not specified
De lara et al. [33]	2013	GTR	Visual function has fully recovered	Transient diabetes insipidus	0%	Not specified	No recurrence
Iacoangeli et al. [34]	2014	GTR	Visual and endocrine deficits rapidly improved	Panhypopituitarism and diabetes insipidus.	0%	2 years	No recurrence
Ceylan et al. [35]	2015	10 GTR	8 patients’ visual deficit and/or visual acuity defects (7 had improvement)	2 patients menstrual cycle disorders, 8 patients’ various endocrine disorders	2 of 16 (12.5%)	2–89 months	Not specified
Sankhla et al. [36]	2015	10 GTR, 4 STR, 1 PR	9 diminished vision, vision recovered in 77.3%	3 patients with diabetes insipidus and 2 with panhypopituitarism	3 of 15 (20%)	20 months to 6 years	Not specified
Javadpour et al. [38]	2016	GTR 3, NTR 3, STR 4, Debulking 1	Preop 9 visual deficit, postop 7 improved	Hypopituitarism and diabetes insipidus (vast majority)	12 of 136 (8.8%)	3 months to 4 years	No recurrence
Fomichev et al. [37]	2016	GTR (72%),	Improved (89%), decrease (11%),	New or worsening hypothalamic-pituitary dysfunction 42.6%	2 patients	Mean follow-up 42 months	27 patients (20%)
Nishioka et al. [39]	2016	Case 1: GTR Case 2: GTR Case 3: GTR	Case 1: improved Case 2: improved Case 3: improved	Case 1: panhypopituitarism, DI Case 2: panhypopituitarism, DI Case 3: panhypopituitarism, DI	1 patient	Case 1: 14 months Case 2: 11 months Case 3: 30 months	Case 1: no case 2: no case 3: no
Wannemuehler et al. [40]	2016	GTR: 5 (55.5), STR: 4 (44.4)	Improved: 8 (88.9), stable: 1 (11.1)	Panhypopituitarism: 3 (33.3), permanent di: 5 (55.5)	2 of 9 (22.2%)	Mean follow-up time in days (SD): 216 (178.9)	1 patient
Alalade et al. [41]	2018	5 (45%) GTR, 2 (18%) NTR, 3 (27%) STR.	Vision stable or improved 8 (73%), vision worsened: 1	New-onset hypopituitarism 6, New thyroid dysfunction 5, growth hormone dysfunction 1, DI: 6 (54%)	1 of 11 (9%)	More than 36 months, 4 patients was less than 36 months	Recurrence 1 patient after a mean follow-up of 43 months
Almedia et al. [42]	2018	Not specified	Not specified	Not specified	Not specified	Not specified	Not specified
Liu et al. [43]	2018	GTR	Improved	Improved	Not specified	Not specified	Not specified
Gomes et al. [44]	2018	Not specified	Visual improvement	Panhypopituitarism on long-term follow-up	Not specified	Not specified	Not specified
Messerer et al. [45]	2018	GTR	Bitemporal hemianopia regressed and the visual acuity improved. A novel left homonymous hemianopia developed secondary to optic tract manipulation.	Not specified	Not specified	Not specified	Not specified
Sweeney et al. [46]	2017	GTR: 5, STR: 5, Debulking: 1	Improved 7 patients	Hypopituitarism and DI on follow-up	2 of 12 (16.7%)	Not specified	2 patients have required further surgery for tumor progression following initial STR.
Todeschini et al. [47]	2017	NTR	At 3-month follow-up, his vision was back to normal	Panhypopituitarism 2 years after radiation therapy	Not specified	6 years	No signs of recurrence
Gauden et al. [48]	2019	GTR 89%, STR 11%	Improvement deficit 44%, No resolution 56%	New panhypopituitarism 20%, Established Postoperative DI. 67%	1 patient	44 months	1patient repeat endoscopic resection followed by radiotherapy 12 months after the initial presentation, 2 patients repeat endoscopic surgery
Wang et al. [49]	2019	NTR	Improved outcome (vision) at 1 year	Transient DI + mild hypernatremia, resolved before discharge; preop endocrine normal	0%	1 year	No recurrence at 1-year MRI
Almeida et al. [50]	2019	NTR	Visual improvement	No preop hormonal deficits; postop new DI/deficits not reported (discharged day 5 “no complications”)	0%	Not reported	Not reported
Liu et al. [51]	2020	NTR	Improved to 20/20 on the right and 20/25 on the left with resolution of bitemporal hemianopsia.	There was transient diabetes insipidus which resolved after a few doses of DDAVP.	0%	3.5 years	No
Tosaka et al. [52]	2020	GTR 81% (17/21); NTR 90% resection in 3; PR1	Improved 11; unchanged 6; deteriorated 3	All patients needed some endocrinological compensation (specific DI/new deficits not quantified)	1 patient	Not reported	Reoperation for recurrence: 2; small recurrences: 4 treated with stereotactic radiotherapy
Javadpour et al. [53]	2021	GTR 4/15; NTR 5/15; STR 6/15	VF normalized in 9/13 with preop VF defects; 1 new persistent VF defect	New anterior pituitary dysfunction 6/11; new DI 9/12 (among those without preop DI); at last follow-up 14/15 anterior panhypopituitarism and 13/15 DI	0%	Follow-up range 8–104 months; median reported 74 months in abstract (also detailed FU stats in Results)	Progression in 2/15, both after initial STR without RT, requiring further surgery/therapy
Ryan et al. [54]	2021	GTR	Not reported	Not reported	0%	6 months	No recurrence at 6 months
Ohta et al. [55]	2022	GTR	Not reported	Postoperative central DI (diagnosed on day of surgery) + hypopituitarism (pituitary function lost)	0%	Discharged post-op day 79	Not reported
Guk and Chukov [56]	2023	GTR: 50.7%; STR: 31.9%; PR: 13%	66.7% Improved (of those with pre-op impairment), 13% Stable, 18.5% Deteriorated	New permanent DI: 33.3%; New hypopituitarism: 26.1%	8 of 69 (11.6%)	Dynamic catamnesis follow-up visits	High recurrence rate noted as a general challenge (specific cohort rate not specified)
Kamal et al. [57]	2023	Debulking	Improved visual fields	New central diabetes insipidus (chronic)	0%	3 months	Yes (this was a redo surgery for cystic recurrence)
Khalil et al. [58]	2023	GTR	Improved visual field	Pre-existing panhypopituitarism and DI; post-op DI worsened	0%	Not specified	Not specified
Shen et al. [59]	2023	GTR	Preserved	Preserved hypothalamic functions	0%	Not specified	Not specified
Constanzo et al. [60]	2024	GTR	Improved	Developed Diabetes Insipidus; No other hypothalamic dysfunction	Not specified	Not specified	Previous recurrence (status post-transcranial surgery)
Chen et al. [61]	2024	GTR	Recovered (Visual acuity improved postoperatively)	Pre-op decreased thyroid hormone; post-op preservation of pituitary stalk for endocrine protection	0%	6 months	No (No recurrence shown on 6-month MRI)
Vigo et al. [62]	2023	GTR	Excellent recovery	Stalk preserved; specific post-op deficits not listed	Not specified	Not specified	Not specified
Eaton et al. [63]	2024	GTR	No new visual deficits; improved fundoscopic exam	New panhypopituitarism with diabetes insipidus (DI)	0%	6 months	No evidence of recurrence
Finger et al. [64]	2024	NTR	Improved	Persistent DI and central hypothyroidism	Not specified	2 months	Not specified
Matmusayev et al. [65]	2024	GTR	Improved immediately	Anterior function preserved; Mild post-op DI	0%	19 months	No
Moiyadi et al. [66]	2024	GTR	Improved	Pre-op panhypopituitarism; post-op transient DI and persistent deficits	0%	4 years	No
Noiphithak et al. [67]	2024	GTR	Fully resolved (at 1 month)	Transient DI; All preoperative deficits resolved at 1 month	Not specified	1 month	Not specified
Bove et al. [68]	2025	GTR: 65.6%; NTR: 13.1%	76.9% improved, 19.2% stable, 3.9% worsened	New DI: 74.1%; New hypopituitarism: 86.7%	6 of 61 (9.8%)	Mean 79.13 months	6.5% (4/61)
Matmusaev et al. [69]	2025	GTR	Significant improvement in visual disturbances following surgery (visus: OD-1.0; OS-1.0)	Managed with hormone replacement therapy; preoperative hypopituitarism with DI	0%	15 months	No
Olson et al. [70]	2025	GTR	Stable vision at one-year postoperatively	Postoperative diabetes insipidus managed with DDAVP	Not specified	12 months	No
Elshazly et al. [71]	2026	GTR: 35.7% (n = 5); NTR: 35.7% (n = 5)	57.1% improved; 28.6% stable; 14.3% worsened	New DI: 85.7% (n = 12); New anterior deficits: 35.7% (n = 5)	1 of 14 (7.1%)	28.3 months (Range: 12–45)	No
Andrade et al. [72]	2023	Not specified	Not specified	Not specified	6.6%	Not specified	Not specified

CSF: Cerebrospinal Fluid; DDAVP: Desmopressin (1-deamino-8-D-arginine vasopressin); DI: Diabetes Insipidus; GTR: Gross Total Resection; MRI: Magnetic Resonance Imaging; NTR: Near Total Resection; OD: Oculus Dexter (Right Eye); OS: Oculus Sinister (Left Eye); RT: Radiotherapy; SD: Standard Deviation; STR: Subtotal Resection; VF: Visual Field.

## Data Availability

The datasets generated during and/or analyzed during the current study are available from the corresponding author on reasonable request.

## Supplementary Materials

The following supporting information can be downloaded at: https://www.mdpi.com/article/10.3390/jcm15083072/s1. Table S1. The PRISMA 2020 checklist. Table S2. Database-Specific Search Strings and Total Number of Results. Table S3: Quality Assessment Tool for Observational Cohort and Cross-Sectional Studies (NIH). Table S4: JBI Critical Appraisal Checklist for Case Reports. Table S5: JBI Critical Appraisal Checklist for Case Series.
