# Supplementary material for: The Evolving Role of the Endoscopic Endonasal Transplanum–Transtuberculum Approach in the Management of Craniopharyngiomas: A Systematic Review of Outcomes, Reconstruction, and Surgical Evolution
Source: J Clin Med. 2026 Apr 17;15(8):3072. doi: 10.3390/jcm15083072 (PMC13117643; doi:10.3390/jcm15083072)
Supplement: Supplementary file 1 [file jcm-15-03072-s001.zip › jcm-4202248-supplementary.pdf]

Table S1. The PRISMA 2020 checklist

Section and Topic	Item #	Checklist item	Location where item is reported
**TITLE**			
Title	1	Identify the report as a systematic review.	1
**ABSTRACT**			
Abstract	2	See the PRISMA 2020 for Abstracts checklist.	2
**INTRODUCTION**			
Rationale	3	Describe the rationale for the review in the context of existing knowledge.	3-4
Objectives	4	Provide an explicit statement of the objective(s) or question(s) the review addresses.	3-4
**METHODS**			
Eligibility criteria	5	Specify the inclusion and exclusion criteria for the review and how studies were grouped for the syntheses.	4
Information sources	6	Specify all databases, registers, websites, organisations, reference lists and other sources searched or consulted to identify studies. Specify the date when each source was last searched or consulted.	4
Search strategy	7	Present the full search strategies for all databases, registers and websites, including any filters and limits used.	4
Selection process	8	Specify the methods used to decide whether a study met the inclusion criteria of the review, including how many reviewers screened each record and each report retrieved, whether they worked independently, and if applicable, details of automation tools used in the process.	5
Data collection process	9	Specify the methods used to collect data from reports, including how many reviewers collected data from each report, whether they worked independently, any processes for obtaining or confirming data from study investigators, and if applicable, details of automation tools used in the process.	5
Data items	10a	List and define all outcomes for which data were sought. Specify whether all results that were compatible with each outcome domain in each study were sought (e.g. for all measures, time points, analyses), and if not, the methods used to decide which results to collect.	5
	10b	List and define all other variables for which data were sought (e.g. participant and intervention characteristics, funding sources). Describe any assumptions made about any missing or unclear information.	5
Study risk of bias assessment	11	Specify the methods used to assess risk of bias in the included studies, including details of the tool(s) used, how many reviewers assessed each study and whether they worked independently, and if applicable, details of automation tools used in the process.	5
Effect measures	12	Specify for each outcome the effect measure(s) (e.g. risk ratio, mean difference) used in the synthesis or presentation of results.	NA
Synthesis methods	13a	Describe the processes used to decide which studies were eligible for each synthesis (e.g. tabulating the study intervention characteristics and comparing against the planned groups for each synthesis (item #5)).	5
	13b	Describe any methods required to prepare the data for presentation or synthesis, such as handling of missing summary statistics, or data conversions.	NA
	13c	Describe any methods used to tabulate or visually display results of individual studies and syntheses.	NA
	13d	Describe any methods used to synthesize results and provide a rationale for the choice(s). If meta-analysis was performed, describe the model(s), method(s) to identify the presence and extent of statistical heterogeneity, and software package(s) used.	NA
	13e	Describe any methods used to explore possible causes of heterogeneity among study results (e.g. subgroup analysis, meta-regression).	NA
	13f	Describe any sensitivity analyses conducted to assess robustness of the synthesized results.	NA
Reporting bias assessment	14	Describe any methods used to assess risk of bias due to missing results in a synthesis (arising from reporting biases).	5
Certainty assessment	15	Describe any methods used to assess certainty (or confidence) in the body of evidence for an outcome.	5
**RESULTS**			

Section and Topic	Item #	Checklist item	Location where item is reported
Study selection	16a	Describe the results of the search and selection process, from the number of records identified in the search to the number of studies included in the review, ideally using a flow diagram.	6
	16b	Cite studies that might appear to meet the inclusion criteria, but which were excluded, and explain why they were excluded.	6
Study characteristics	17	Cite each included study and present its characteristics.	7
Risk of bias in studies	18	Present assessments of risk of bias for each included study.	7
Results of individual studies	19	For all outcomes, present, for each study: (a) summary statistics for each group (where appropriate) and (b) an effect estimate and its precision (e.g. confidence/credible interval), ideally using structured tables or plots.	7
Results of syntheses	20a	For each synthesis, briefly summarise the characteristics and risk of bias among contributing studies.	6-8
	20b	Present results of all statistical syntheses conducted. If meta-analysis was done, present for each the summary estimate and its precision (e.g. confidence/credible interval) and measures of statistical heterogeneity. If comparing groups, describe the direction of the effect.	6-8
	20c	Present results of all investigations of possible causes of heterogeneity among study results.	6-8
	20d	Present results of all sensitivity analyses conducted to assess the robustness of the synthesized results.	6-8
Reporting biases	21	Present assessments of risk of bias due to missing results (arising from reporting biases) for each synthesis assessed.	6-8
Certainty of evidence	22	Present assessments of certainty (or confidence) in the body of evidence for each outcome assessed.	6-8
**DISCUSSION**			
Discussion	23a	Provide a general interpretation of the results in the context of other evidence.	9-14
	23b	Discuss any limitations of the evidence included in the review.	9-14
	23c	Discuss any limitations of the review processes used.	9-14
	23d	Discuss implications of the results for practice, policy, and future research.	9-14
**OTHER INFORMATION**			
Registration and protocol	24a	Provide registration information for the review, including register name and registration number, or state that the review was not registered.	4
	24b	Indicate where the review protocol can be accessed, or state that a protocol was not prepared.	4
	24c	Describe and explain any amendments to information provided at registration or in the protocol.	4
Support	25	Describe sources of financial or non-financial support for the review, and the role of the funders or sponsors in the review.	1
Competing interests	26	Declare any competing interests of review authors.	1
Availability of data, code and other materials	27	Report which of the following are publicly available and where they can be found: template data collection forms; data extracted from included studies; data used for all analyses; analytic code; any other materials used in the review.	1

Table S2. Database-Specific Search Strings and Total Number of Results

The Role and Outcomes of the Endoscopic Endonasal Transplanum–Transtuberculum Approach in Craniopharyngioma Management: A Systematic Review and Technical Classification<br>***We searched online databases: Medline (through PubMed), Scopus, Embase, and Web of Science up to January 21, 2026.***		
Term:<br>***#1= ("Craniopharyngioma" OR "Craniopharyngiomas" OR "Craniopharyngeal Tumor" OR "Craniopharyngeal Tumors" OR "Adamantinous Craniopharyngioma" OR "Adamantinous Craniopharyngiomas" OR "Papillary Craniopharyngioma" OR "Papillary Craniopharyngiomas" OR "Adult Craniopharyngioma" OR "Adult Craniopharyngiomas" OR "Craniopharyngioma, Child" OR "Child Craniopharyngioma" OR "Child Craniopharyngiomas"")***<br><br>***#2= ("Endoscopic Endonasal" OR "Endonasal Endoscopic" OR "Endoscopic Endonasal Approach" OR "Endonasal Endoscopic Approach" OR "Endoscopic Endonasal Surgery" OR "Endonasal Endoscopic Surgery" OR "Endoscopic Endonasal Skull Base Surgery" OR "Endoscopic Endonasal Transsphenoidal" OR "Endonasal Endoscopic Transsphenoidal")***<br>***#3=("Transplanum" OR "Transplanum Sphenoidale" OR "Planum Sphenoidale" OR "Transplanar" OR "Transtuberculum" OR "Transtubercular" OR "Tuberculum Sellae" OR "Trans-Tuberculum" OR "Suprasellar Transtuberculum" OR "Transplanum Transtuberculum Approach")***<br><br>***#4=1 AND 2 AND 3***		
**Database**	*Search Term*	*Number of articles*
Scopus	*TITLE-ABS(("Craniopharyngioma" OR "Craniopharyngiomas" OR "Craniopharyngeal Tumor" OR "Craniopharyngeal Tumors" OR "Adamantinous Craniopharyngioma" OR "Adamantinous Craniopharyngiomas" OR "Papillary Craniopharyngioma" OR "Papillary Craniopharyngiomas" OR "Adult Craniopharyngioma" OR "Adult Craniopharyngiomas" OR "Craniopharyngioma, Child" OR "Child Craniopharyngioma" OR "Child Craniopharyngiomas") AND ("Endoscopic*	*95*

	Endonasal" OR "Endonasal Endoscopic" OR "Endoscopic Endonasal Approach" OR "Endonasal Endoscopic Approach" OR "Endoscopic Endonasal Surgery" OR "Endonasal Endoscopic Surgery" OR "Endoscopic Endonasal Skull Base Surgery" OR "Endoscopic Endonasal Transsphenoidal" OR "Endonasal Endoscopic Transsphenoidal") AND ("Transplanum" OR "Transplanum Sphenoidale" OR "Planum Sphenoidale" OR "Transplanar" OR "Transtuberculum" OR "Transtubercular" OR "Tuberculum Sellae" OR "Trans-Tuberculum" OR "Suprasellar Transtuberculum" OR "Transplanum Transtuberculum Approach"))	
Web of Science	*TS=(("Craniopharyngioma" OR "Craniopharyngiomas" OR "Craniopharyngeal Tumor" OR "Craniopharyngeal Tumors" OR "Adamantinous Craniopharyngioma" OR "Adamantinous Craniopharyngiomas" OR "Papillary Craniopharyngioma" OR "Papillary Craniopharyngiomas" OR "Adult Craniopharyngioma" OR "Adult Craniopharyngiomas" OR "Craniopharyngioma, Child" OR "Child Craniopharyngioma" OR "Child Craniopharyngiomas") AND ("Endoscopic Endonasal" OR "Endonasal Endoscopic" OR "Endoscopic Endonasal Approach" OR "Endonasal Endoscopic Approach" OR "Endoscopic Endonasal Surgery" OR "Endonasal Endoscopic Surgery" OR "Endoscopic Endonasal Skull Base Surgery" OR "Endoscopic Endonasal Transsphenoidal" OR "Endonasal Endoscopic Transsphenoidal") AND ("Transplanum" OR "Transplanum Sphenoidale" OR "Planum Sphenoidale" OR "Transplanar" OR "Transtuberculum" OR "Transtubercular" OR "Tuberculum Sellae" OR "Trans-Tuberculum" OR "Suprasellar Transtuberculum" OR "Transplanum Transtuberculum Approach"))*	*71*
PubMed	*(("Craniopharyngioma"[tiab] OR "Craniopharyngiomas"[tiab] OR "Craniopharyngeal Tumor"[tiab] OR "Craniopharyngeal Tumors"[tiab] OR "Adamantinous Craniopharyngioma"[tiab] OR "Adamantinous Craniopharyngiomas"[tiab] OR "Papillary Craniopharyngioma"[tiab] OR "Papillary Craniopharyngiomas"[tiab] OR "Adult Craniopharyngioma"[tiab] OR "Adult Craniopharyngiomas"[tiab] OR "Craniopharyngioma, Child"[tiab] OR "Child Craniopharyngioma"[tiab] OR "Child Craniopharyngiomas"[tiab]) AND ("Endoscopic*	*78*

	Endonasal"[tiab] OR "Endonasal Endoscopic"[tiab] OR "Endoscopic Endonasal Approach"[tiab] OR "Endonasal Endoscopic Approach"[tiab] OR "Endoscopic Endonasal Surgery"[tiab] OR "Endonasal Endoscopic Surgery"[tiab] OR "Endoscopic Endonasal Skull Base Surgery"[tiab] OR "Endoscopic Endonasal Transsphenoidal"[tiab] OR "Endonasal Endoscopic Transsphenoidal"[tiab]) AND ("Transplanum"[tiab] OR "Transplanum Sphenoidale"[tiab] OR "Planum Sphenoidale"[tiab] OR "Transplanar"[tiab] OR "Transtuberculum"[tiab] OR "Transtubercular"[tiab] OR "Tuberculum Sellae"[tiab] OR "Trans-Tuberculum"[tiab] OR "Suprasellar Transtuberculum"[tiab] OR "Transplanum Transtuberculum Approach"[tiab]))	
Embase	*(('Craniopharyngioma':ti,ab OR 'Craniopharyngiomas':ti,ab OR 'Craniopharyngeal Tumor':ti,ab OR 'Craniopharyngeal Tumors':ti,ab OR 'Adamantinous Craniopharyngioma':ti,ab OR 'Adamantinous Craniopharyngiomas':ti,ab OR 'Papillary Craniopharyngioma':ti,ab OR 'Papillary Craniopharyngiomas':ti,ab OR 'Adult Craniopharyngioma':ti,ab OR 'Adult Craniopharyngiomas':ti,ab OR 'Craniopharyngioma, Child':ti,ab OR 'Child Craniopharyngioma':ti,ab OR 'Child Craniopharyngiomas':ti,ab) AND ('Endoscopic Endonasal':ti,ab OR 'Endonasal Endoscopic':ti,ab OR 'Endoscopic Endonasal Approach':ti,ab OR 'Endonasal Endoscopic Approach':ti,ab OR 'Endoscopic Endonasal Surgery':ti,ab OR 'Endonasal Endoscopic Surgery':ti,ab OR 'Endoscopic Endonasal Skull Base Surgery':ti,ab OR 'Endoscopic Endonasal Transsphenoidal':ti,ab OR 'Endonasal Endoscopic Transsphenoidal':ti,ab) AND ('Transplanum':ti,ab OR 'Transplanum Sphenoidale':ti,ab OR 'Planum Sphenoidale':ti,ab OR 'Transplanar':ti,ab OR 'Transtuberculum':ti,ab OR 'Transtubercular':ti,ab OR 'Tuberculum Sellae':ti,ab OR 'Trans-Tuberculum':ti,ab OR 'Suprasellar Transtuberculum':ti,ab OR 'Transplanum Transtuberculum Approach':ti,ab))*	*81*
Total	***325***	

Table S3: Quality Assessment Tool for Observational Cohort and Cross-Sectional Studies (NIH)

Study	Q1	Q2	Q3	Q4	Q5	Q6	Q7	Q8	Q9	Q10	Q11	Q12	Q13	Q14	Overall rating
Ceylan et al. [27]	+	+	+	+	NA	+	+	NA	+	NA	+	NA	+	+	**High Quality**
Fomichev et al. [37]	+	+	+	+	NA	+	+	+	+	+	+	NA	+	+	**High Quality**
Javadpour et al. [38]	+	+	+	+	NA	+	+	+	NA	-	+	NA	NA	NA	**Fair Quality**
Wannemuehler et al. [40]	+	+	+	+	NA	+	+	NA	+	-	+	NA	+	+	**Fair Quality**
Alalade et al. [41]	+	+	+	+	NA	+	+	+	+	+	+	NA	+	+	**High Quality**
Sweeney et al. [46]	+	+	+	+	NA	+	+	+	NA	-	+	NA	NA	NA	**Fair Quality**
Gauden et al. [48]	+	+	+	+	NA	+	+	+	+	+	+	NA	+	+	**High Quality**
Javadpour et al. [53]	+	+	+	+	-	+	+	+	+	-	+	NA	+	+	**High Quality**
Guk et al. [56]	+	+	+	+	NA	+	+	NA	+	NA	+	NA	+	+	**High Quality**
Bove et al. [68]	+	+	+	+	NA	+	+	+	+	-	+	NA	+	+	**High Quality**
Elshazly et al. [71]	+	+	+	+	NA	+	+	+	+	+	+	NA	+	+	**High Quality**
Andrade et al. [72]	+	+	+	+	NA	+	+	+	NA	-	+	NA	NA	NA	**Fair Quality**

Quality Assessment Tool for Observational Cohort and Cross-Sectional Studies (NIH)
(+): Yes/Low risk, (-): No/High risk, (*): Unclear, NA: Not applicable

Q1: Was the research question or objective in this paper clearly stated?

Q2: Was the study population clearly specified and defined?

Q3: Was the participation rate of eligible persons at least 50%?

Q4: Were all the subjects selected or recruited from the same or similar populations (including the same time period)? Were inclusion and exclusion criteria for being in the study prespecified and applied uniformly to all participants?

Q5: Was a sample size justification, power description, or variance and effect estimates provided?

Q6: For the analyses in this paper, were the exposure(s) of interest measured prior to the outcome(s) being measured?

Q7: Was the timeframe sufficient so that one could reasonably expect to see an association between exposure and outcome if it existed?

Q8: For exposures that can vary in amount or level, did the study examine different levels of the exposure as related to the outcome (e.g., categories of exposure, or exposure measured as continuous variable)?

Q9: Were the exposure measures (independent variables) clearly defined, valid, reliable, and implemented consistently across all study participants?

Q10: Was the exposure(s) assessed more than once over time?

Q11: Were the outcome measures (dependent variables) clearly defined, valid, reliable, and implemented consistently across all study participants?

Q12: Were the outcome assessors blinded to the exposure status of participants?

Q13: Was loss to follow-up after baseline 20% or less?

Q14: Were key potential confounding variables measured and adjusted statistically for their impact on the relationship between exposure(s) and outcome(s)?

Table S4: JBI Critical Appraisal Checklist for Case Reports

References	Q1	Q2	Q3	Q4	Q5	Q6	Q7	Q8	Overall rating
Liu et al. [51]	+	+	+	+	+	+	+	+	**High Quality**
De Divitiis et al. [12]	+	+	+	+	+	+	+	+	**High Quality**
Laufer et al. [25]	+	+	+	+	+	+	+	+	**High Quality**
Cavallo et al. [26]	+	+	+	-	+	+	+	+	**High Quality**
Saeki et al. [28]	+	+	+	+	+	+	+	+	**High Quality**
Ferroli et al. [30]	+	+	+	NA	-	+	+	+	**Fair Quality**
Kenning et al. [31]	+	+	+	+	+	+	NA	+	**Fair Quality**
Iacoangeli et al. [34]	+	+	+	+	+	+	+	+	**High Quality**
De Lara et al. [33]	+	+	+	+	+	+	+	+	**High Quality**
Nishioka et al. [39]	+	+	+	+	+	+	+	+	**High Quality**
Liu et al. [32]	-	-	-	+	+	-	-	+	**Low Quality**
Almeida et al. [42]	+	+	+	+	+	+	+	-	**High Quality**
Liu et al. [43]	+	+	+	+	+	+	NA	+	**High Quality**
Mangussi-Gomes et al. [44]	+	+	+	+	+	+	+	+	**High Quality**
Messerer et al. [45]	+	+	+	+	+	+	+	+	**High Quality**
Todeschini et al. [47]	+	+	+	+	+	+	+	+	**High Quality**
Wang et al. [49]	+	+	+	+	+	+	+	+	**High Quality**
Almeida et al. [50]	+	+	+	+	+	+	+	+	**High Quality**
Ryan et al. [54]	+	+	+	+	+	+	NA	+	**High Quality**
Ohta et al. [55]	+	+	+	+	+	+	+	+	**High Quality**
Kamal et al. [57]	+	+	+	+	+	+	+	+	**High Quality**
Khalil et al. [58]	+	+	+	+	+	+	NA	+	**High Quality**

Shen et al. [59]	+	+	+	+	+	+	NA	+	**High Quality**
Chen et al. [61]	+	+	+	+	+	+	+	+	**High Quality**
Vigo et al. [62]	+	+	+	+	+	+	+	+	**High Quality**
Constanzo et al. [60]	+	+	+	+	+	+	+	+	**High Quality**
Eaton et al. [63]	+	+	+	+	+	+	+	+	**High Quality**
Finger et al. [64]	+	+	+	+	+	+	+	+	**High Quality**
Matmusayev et al. [65]	+	+	+	+	+	+	+	+	**High Quality**
Moiyadi et al. [66]	+	+	+	+	+	+	+	+	**High Quality**
Noiphithak et al. [67]	+	+	+	+	+	+	+	-	**High Quality**
Matmusaev et al. [69]	+	+	+	+	+	+	+	+	**High Quality**
Olson et al. [70]	+	+	+	+	+	+	+	+	**High Quality**

(+): Yes, (-): No, (*): Unclear, Not/Applicable (NA)

Q1. Were patient's demographic characteristics clearly described?

Does the case report clearly describe patient's age, sex, race, medical history, diagnosis, prognosis, previous treatments, past and current diagnostic test results, and medications? The setting and context may also be described.

Q2. Was the patient's history clearly described and presented as a timeline?

A good case report will clearly describe the history of the patient, their medical, family and psychosocial history including relevant genetic information, as well as relevant past interventions and their outcomes.

Q3. Was the current clinical condition of the patient on presentation clearly described?

The current clinical condition of the patient should be described in detail including the uniqueness of the condition/disease, symptoms, frequency and severity. The case report should also be able to present whether differential diagnoses was considered.

Q4. Were diagnostic tests or methods and the results clearly described?

A reader of the case report should be provided sufficient information to understand how the patient was assessed. It is important that all appropriate tests are ordered to confirm a diagnosis and thereforethe case report should provide a clear description of various diagnostic tests used (whether a gold standard or alternative diagnostic tests). Photographs or illustrations of diagnostic procedures, radiographs, or treatment procedures are usually presented when appropriate to convey a clear message to readers

Q5. Was the intervention(s) or treatment procedure(s) clearly described?

It is important to clearly describe treatment or intervention procedures as other clinicians will be reading the paper and therefore may enable clear understanding of the treatment protocol. The report should describe the treatment/intervention protocol in detail; for e.g. in pharmacological management of dental anxiety - the type of drug, route of administration, drug dosage and frequency, and any sideeffects.

Q6. Was the post-intervention clinical condition clearly described?

A good case report should clearly describe the clinical condition post-intervention in terms of the presence or lack thereof symptoms. The outcomes of management/treatment when presented asimages or figures would help in conveying the information to the reader/clinician.

Q7. Were adverse events (harms) or unanticipated events identified and described?

With any treatment/intervention/drug, there are bound to be some adverse events and, in some cases, they may be severe. It is important that adverse events are clearly documented and described, particularly when a new or unique condition is being treated or when a new drug or treatment is used. In addition, unanticipated events, if any that may yield new or useful information should be identified and clearly described.

Q8. Does the case report provide takeaway lessons?

Case reports should summarize key lessons learned from a case in terms of the background of the condition/disease and clinical practice guidance for clinicians when presented with similar cases.

Table S5: JBI Critical Appraisal Checklist for Case Series

References	Q1	Q2	Q3	Q4	Q5	Q6	Q7	Q8	Q9	Q10	Overall rating
Frank et al. [23]	+	+	+	+	+	+	+	+	+	+	High Quality
Divitiis et al. [24]	+	+	+	+	+	+	+	+	+	-	High Quality
Kalinin et al. [29]	+	+	+	+	NA	-	+	+	+	-	Low Quality
Tosaka et al. [52]	+	+	+	+	+	+	+	+	+	NA	High Quality
Ceylan et al. [35]	+	+	+	+	+	+	+	+	+	NA	High Quality
Sankhla et al. [36]	+	+	+	+	+	+	+	+	+	NA	High Quality

(+): Yes, (-): No, (*): Unclear, Not/Applicable (NA)

Q1. Were there clear criteria for inclusion in the case series?

The authors should provide clear inclusion (and exclusion criteria where appropriate) for the study participants. The inclusion/exclusion criteria should be specified (e.g., risk, stage of disease progression) with sufficient detail and all the necessary information critical to the study.

Q2. Was the condition measured in a standard, reliable way for all participants included in the case series?

The study should clearly describe the method of measurement of the condition. This should be done in a standard (i.e. same way for all patients) and reliable (i.e. repeatable and reproducible results) way.

Q3. Were valid methods used for identification of the condition for all participants included in the case series?

Many health problems are not easily diagnosed or defined and some measures may not be capable of including or excluding appropriate levels or stages of the health problem. If the outcomes were assessed based on existing definitions or diagnostic criteria, then the answer to this question is likely to be yes. If the outcomes were assessed using observer reported, or self-reported scales, the risk of over- or under-reporting is increased, and objectivity is compromised. Importantly, determine if the measurement tools used were validated instruments as this has a significant impact on outcome assessment validity.

Q4. Did the case series have consecutive inclusion of participants?

Studies that indicate a consecutive inclusion are more reliable than those that do not. For example, a case series that states 'we included all patients (24) with osteosarcoma who presented to our clinic between March 2005 and June 2006' is more reliable than a study that simply states 'we report a case series of 24 people with osteosarcoma.'

Q5. Did the case series have complete inclusion of participants?

The completeness of a case series contributes to its reliability (1). Studies that indicate a complete inclusion are more reliable than those that do not. A stated above, a case series that states 'we included all patients (24) with osteosarcoma who presented to our clinic between March 2005 and June 2006' is more reliable than a study that simply states 'we report a case series of 24 people with osteosarcoma.'

Q6. Was there clear reporting of the demographics of the participants in the study?

The case series should clearly describe relevant participant's demographics such as the following information where relevant: participant's age, sex, education, geographic region, ethnicity, time period, education.

Q7. Was there clear reporting of clinical information of the participants?

There should be clear reporting of clinical information of the participants such as the following information where relevant: disease status, comorbidities, stage of disease, previous interventions/treatment, results of diagnostic tests, etc.

Q8. Were the outcomes or follow-up results of cases clearly reported?

The results of any intervention or treatment should be clearly reported in the case series. A good case study should clearly describe the clinical condition post-intervention in terms of the presence or lack of symptoms. The outcomes of management/treatment when presented as images or figures can help in conveying the information to the reader/clinician. It is important that adverse events are clearly documented and described, particularly a new or unique condition is being treated or when a new drug or treatment is used. In addition, unanticipated events, if any that may yield new or useful information should be identified and clearly described.

Q9. Was there clear reporting of the presenting site(s)/clinic(s) demographic information?

Certain diseases or conditions vary in prevalence across different geographic regions and populations (e.g. women vs. men, sociodemographic variables between countries). The study sample should be described in sufficient detail so that other researchers can determine if it is comparable to the population of interest to them.

Q10. Was statistical analysis appropriate?

As with any consideration of statistical analysis, consideration should be given to whether there was a more appropriate alternate statistical method that could have been used. The methods section of studies should be detailed enough for reviewers to identify which analytical techniques were used and whether these were suitable.
